# Food Network Analysis in Non-Obese Patients with or without Steatosis

**DOI:** 10.3390/nu15122713

**Published:** 2023-06-11

**Authors:** Rossella Donghia, Pasqua Letizia Pesole, Sergio Coletta, Caterina Bonfiglio, Giovanni De Pergola, Sara De Nucci, Roberta Rinaldi, Gianluigi Giannelli

**Affiliations:** National Institute of Gastroenterology—IRCCS “Saverio de Bellis”, 70013 Castellana Grotte, Italy; letizia.pesole@irccsdebellis.it (P.L.P.); sergio.coletta@irccsdebellis.it (S.C.); catia.bonfiglio@irccsdebellis.it (C.B.); giovanni.depergola@irccsdebellis.it (G.D.P.); sara.denucci@irccsdebellis.it (S.D.N.); roberta.rinaldi@irccsdebellis.it (R.R.); gianluigi.giannelli@irccsdebellis.it (G.G.)

**Keywords:** steatosis, BMI, food intake, obesity

## Abstract

Background: Steatosis is the most common liver disease worldwide and the leading cause of liver-associated morbidity and mortality. The aim of this study was to explore the differences in blood parameters and dietary habits in non-obese patients with and without steatosis. Methods: The present study included 987 participants with BMI < 30, assessed in the fourth recall of the MICOL study. Patients were divided by steatosis grade, and a validated food frequency questionnaire (FFQ) with 28 food groups was administered. Results: The prevalence of non-obese participants with steatosis was 42.86%. Overall, the results indicated many statistically significant blood parameters and dietary habits. Analysis of dietary habits revealed that non-obese people with or without steatosis had similar dietary habits, although higher daily amounts of red meat, processed meat, ready meals, and alcohol were recorded in participants with liver disease (*p* < 0.05). Conclusions: Many differences were found in non-obese people with and without steatosis, but in light of a network analysis, the two groups demonstrated similar dietary habits, proving that pathophysiological, genetic, and hormonal patterns are probably the basis of their liver status, regardless of weight. Future genetic analyses will be performed to analyze the expression of genes involved in the development of steatosis in our cohort.

## 1. Introduction

Fatty liver disease is one of the most common chronic liver diseases worldwide [1,2], with a prevalence of approximately 25%. The proportion of patients with more severe liver disease and the incidence of all-cause mortality, liver-related mortality, and cardiovascular mortality among non-obese and obese steatosis patients varies and could be confounded by selection bias, underestimation of alcohol intake, and unaccounted weight changes over time.

The true prevalence of steatosis and its predictors in non-obese individuals are unknown, as are the clinical characteristics and mechanisms of the pathogenesis of this disease in individuals with BMI < 30, as compared to obese individuals [3]. Younes et al. asserted that the prevalence rates of lean/non-obese steatosis vary widely, ranging from 3% to 30% in the world population. This variability may be attributed to several factors, such as patient selection, diagnostic modalities, BMI cut-off values, and lifestyle and dietary customs of the evaluated populations [4]. Several other longitudinal studies have shown conflicting results. Hagström [5] et al. found that patients with lean steatosis were paradoxically more likely to develop severe liver disease, despite having less severe liver disease at baseline, compared to non-lean patients [6,7,8,9]. Further future studies will be necessary to understand the reasons behind these inconsistent findings.

It is well-known that different ethnic groups have different tendencies to accumulate visceral and liver fat and to develop steatosis and metabolic syndrome. A landmark paper by Browning and colleagues [10] was the first to point out ethnic differences in this regard.

Hispanics were found to have the highest prevalence of liver steatosis, while the prevalence was significantly lower among Blacks despite an equally high prevalence of obesity and insulin resistance. In a subsequent multi-ethnic cohort study of 1794 subjects of African, European, Latino, Japanese, and Native Hawaiian ancestry in the United States, the mean visceral and liver fat were highest among the Japanese Americans, which jointly accounted for a statistically significant fraction of the difference in metabolic syndrome prevalence compared to other ethnic groups independently of total fat mass [10,11,12].

The difference in tendency for visceral adiposity, steatosis, and metabolic syndrome between the different ethnic groups may be explained by genetic profiles. A single nucleotide polymorphism in the patatin-like phospholipase domain-containing-3 (*PNPLA3*) gene, the rs738409 variant (C > G), which results in the substitution of isoleucine by methionine at position 148 (I148M), was found to be associated with increased liver fat, and the risk allele was found to be the highest among Hispanics in the cohort and the lowest among Blacks. Genetic polymorphisms in this gene have subsequently been recognized as a major genetic determinant of steatosis and its progression. The *PNPLA3* protein with lipase activity in hepatocytes promotes the accumulation of triglycerides in liver cells [13,14,15]. In another study, the *HSD17B13* rs72613567 and rs6834314 variants were found to be associated with a lower risk of steatosis and adverse liver-related outcomes among Chinese but not Indians and Malay subjects, supporting the role of polygenic determinants in the disease phenotype [16]. The transmembrane 6 superfamily member 2 (*TM6SF2*) encodes a membrane protein required for normal very-low-density lipoprotein secretion. The rs58542926 variant (C > T), which results in the substitution of glutamate by lysine at position 167 (E167K), was found to be associated with higher circulating levels of serum alanine aminotransferase, a biomarker of liver disease, but a lower level of serum low-density lipoprotein cholesterol and triglycerides. In another paper, based on a retrospective cohort of 669 consecutive patients with biopsy-proven steatosis in Italy, a significantly greater proportion of patients with lean steatosis had E167K compared to their non-lean counterparts. In the same study, I148M was the only independent factor found to be associated with steatosis and significant fibrosis among lean patients. Additionally, lean steatosis may be also driven by other rare genetic diseases, such as familial hypobetalipoproteinemia or cholesteryl ester storage disease [17,18,19,20].

Obesity is a major risk because steatosis increases with the BMI, but a substantial proportion of individuals with steatosis can be classified as non-obese (BMI < 30 Kg/m^2^), although the relationship between BMI and steatosis has yet to be clarified [4]. Despite having a normal or lower BMI, lean or non-obese steatosis patients have excess visceral adiposity. Lean or non-obese steatosis patients share a common altered metabolic and cardiovascular profile with their non-lean or obese counterparts, although the alterations are generally less severe [21]. Nevertheless, it is reasonable to think that lean or non-obese steatosis is the early phase of steatosis or the less severe end of the steatosis spectrum.

Steatosis is considered responsible for the majority of chronic liver diseases. Owing to its possible progression to non-alcoholic steatohepatitis (NASH), liver cirrhosis, and hepatocellular carcinoma [22,23], it is vital to understand the physiological causes of steatosis in order to develop personalized treatment plans for the future.

Steatosis develops when the rate of intake of fatty acids and triglycerides is greater than the output. Fatty acids in the liver are derived from plasma-free fatty acids (FFA) released from the hydrolysis of adipose tissue, from triglycerides, and the hydrolysis of circulating, de novo fatty acid synthesis, fatty acid oxidation (FAO), and fatty acid export [24]. Free fatty acids are either stored as triglycerides, exported from the liver, or undergo oxidation. An excess of free fatty acids causes oxidative stress, liver cell injury and death, inflammation, and eventually steatosis and fibrosis.

Steatosis is strongly associated with central adiposity, diabetes, insulin resistance, hypertension, and metabolic syndrome [25,26]. Accumulating evidence indicates that high-calorie diets, especially those rich in saturated and trans fatty acids, and cholesterol, as well as fructose-rich diets, or diets poor in polyunsaturated fatty acids, vitamins, and minerals [27], increase visceral fat [28].

The pathophysiology of lean or non-obese steatosis is not completely understood. Calorie restriction and weight loss are an effective therapy for obese patients with steatosis [29].

It has been demonstrated that diet can also modify the intestinal microbiota, considered an “invisible organ” in the human body that can play an important role in normal metabolism and immuno-modulation [30]. Steatosis has been associated with a lower rate of Bacteroidetes and a higher rate of *Prevotella* and *Porphyromonas*, as well as a higher number of ethanol-producing bacteria [31,32]. Duarte et al. described a significant difference in the abundance of *Faecalibacterium*, *Ruminococcus*, *Lactobacillus*, and *Bifidobacterium* in patients with steatosis when compared with a control group [33]. The subgroup of lean patients with steatosis had less abundance of *Ruminococcus* and a deficiency of *Lactobacilli* when compared with overweight and obese patients with steatosis [34].

Lysophosphatidylcholines (lyso-PCs) are phospholipids with anti-inflammatory and insulin-sensitizing effects; lower levels of lyso-PCs are observed in obesity, and when compared to their obese counterparts, lean patients with steatosis showed a higher level of lysine concentration [35]. Being related to visceral fat accumulation [36], lysine may represent a sign of the dysfunctional metabolic environment underpinning lean steatosis individuals. The impact of the gut microbiota on steatosis has been suggested by previous studies and may be a viable target for future steatosis treatment [37], especially in non-obese steatotic patients.

Lifestyle intervention is the cornerstone for the management of steatosis; however, some papers are based on trials studying whether weight loss is associated with a better prognosis of liver disease only in overweight or obese patients, but not in non-obese subjects [38,39]. The same is the case with multiple drugs targeting obesity and metabolic syndrome promising good results. Liraglutide 1.8 mg daily for 48 weeks or semaglutide have good results on liver parameters but often in patients with BMI > 30.

The purpose of this paper was to explore the differences in the blood parameters and food network intake in non-obese patients, with and without steatosis, in a cohort from southern Italy. To fulfill these objectives, the network of daily food groups intake study was analyzed.

## 2. Materials and Methods

### 2.1. Study Population

Subjects in the present study were recruited for the first time from the electoral register of Castellana Grotte, a town in Southern Italy [40], to participate in a multicenter Italian study on Cholelithiasis (MICOL) [41].

**Figure 1:**
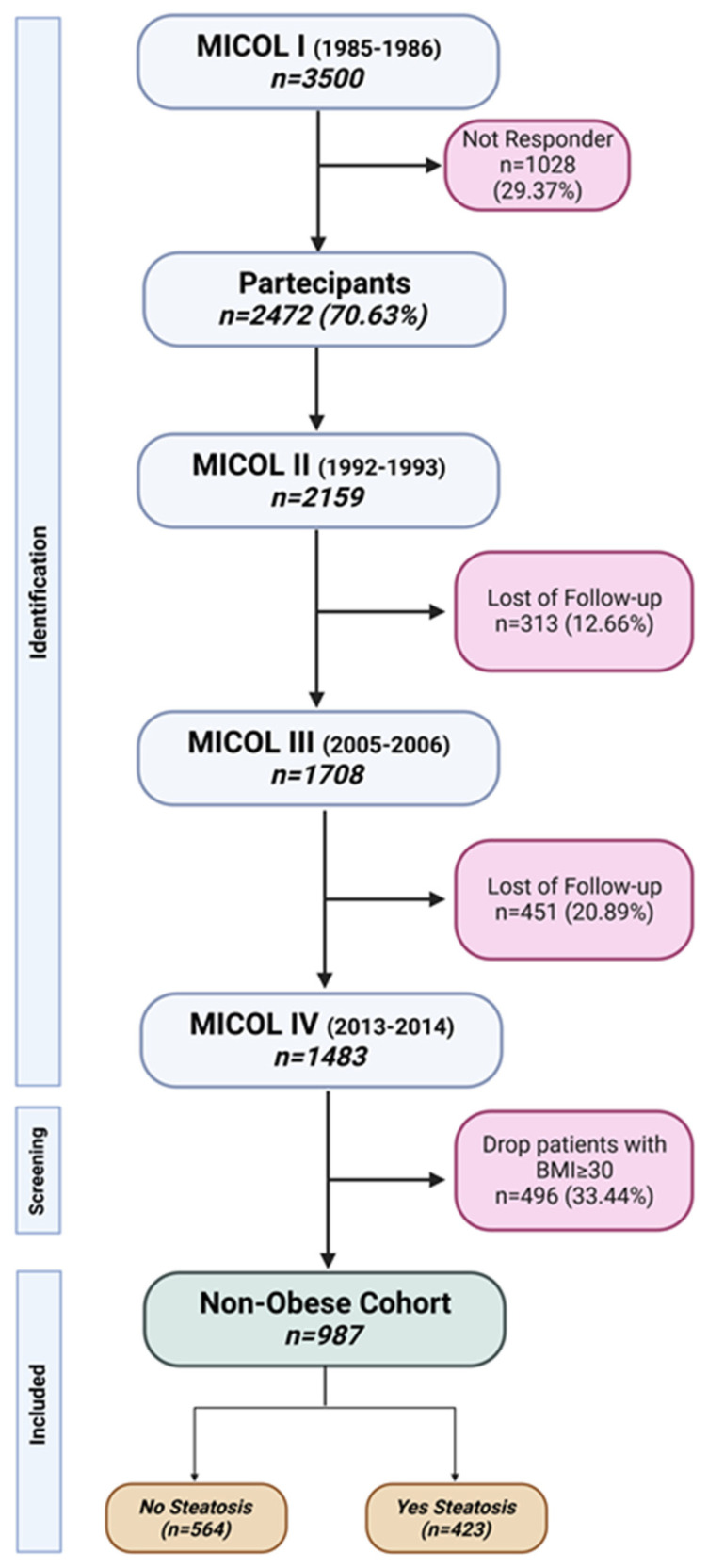
Flowchart of MICOL study and patient selection. This image was created with BioRender (https://app.biorender.com/illustrations/64231d797b9d8ec537d08e06, accessed on 18 April 2023).

The methodological details of this population-based study have been previously published [42,43]. For this study (called MICOL IV), recall of MICOL III was adopted. In the present study, only non-obese patients with BMI < 30 were included, and the steatosis condition was used to split patients into two independent groups (Figure 1).

All participants signed informed consent forms before examination and the study was approved as being in line with the ethical standards of the institutional research committee of the National Institute of Gastroenterology and Research Hospital “S. de Bellis” in Castellana Grotte, Italy (DDG-CE 782/2013). The study was conducted in accordance with the Helsinki Declaration of 1975. The present study adhered to the “Standards for Reporting Diagnostic Accuracy Studies” (STARD) guidelines and the manuscript was organized according to the “Strengthening the Reporting of Observational Studies in Epidemiology–Nutritional Epidemiology” (STROBE-nut) guidelines [44].

The metabolic syndrome variable (MeS) was built based on International Diabetes Federation (IDF) criteria [45], and liver steatosis was established by abdominal ultrasound screening and graded based on liver echogenicity [46].

### 2.2. Dietary Assessments

To evaluate dietary habits, a validated food frequency questionnaire [41,47] was administered during the visit. The questionnaire is organized into 11 sections representing food macro areas: grains, meat, fish, milk and dairy products, vegetables, legumes, fruits, miscellaneous foods, water and alcoholic beverages, olive oil and other edible fats, coffee/sugar, and salt. Each food (86 validated foods) was converted to mean daily intake in grams and the total was summarized in 28 food groups (Supplementary Table S1) established according to similarity type [48].

### 2.3. Statistical Analysis

Non-obese patient characteristics are reported as mean and standard deviation (M ± SD) for continuous variables, and as frequency and percentage (%) for categorical variables. To test the association between the independent groups (Yes Steatosis vs. No Steatosis), the chi-square test was used for categorical variables, where necessary, and the Wilcoxon rank Mann–Whitney test for continuous variables.

To visualize the relationships between food group intakes, a network analysis was performed. Partial correlation corresponds to the degree of association between two variables, controlling for other variables. To visualize these correlations, a network was used which computes a sparse Gaussian graphical model with a graphical lasso [49,50,51]. Two foods were connected by a blue line when they were consumed by the same patients, while red lines represent rare consumption. The bolder the lines, the higher the correlation. The network was generated for the 28 food groups analyzed.

To quantify the importance of each node in the network, we computed centrality indices [52,53]. Centrality indices reflect how potentially clinically relevant is a given node in a network. The “betweenness” centrality is the number of times that a given node acts as a bridge on the shortest path length between any pair of other nodes; “closeness” centrality captures the average distance of a node from all other nodes in the network, computed from the inverse of the weighted sum of shortest path lengths connecting a given node to all the other nodes in the network. “Strength” is the sum of the edge weights attached to that node.

Mean imputation was performed per food group to obtain a complete matrix for network analysis (missing data were less than 20%).

To test the null hypothesis of non-association, the two-tailed probability level was set at 0.05. The analyses were conducted using StataCorp.2021 software (Release 17) College Station, TX: StataCorp LLC., and RStudio (“Prairie Trillium” Release) was used for the graphics.

## 3. Results

As shown in Table 1, the total non-obese cohort consisted of 579 (58.66%) males and 408 (41.34%) females; the prevalence of steatosis was higher in male patients (68.32% vs. 51.42%, *p* < 0.001). The mean age was 63.56 ± 11.57 years, and the most frequent educational level in this cohort was elementary school (31.75%). No significant difference was observed for smoking habit (14.61% vs. 15.53, *p* = 0.79). As expected, the BMI (<30) was statistically significant in each group, with higher levels in steatosis patients (26.72 ± 2.37 vs. 24.84 ± 2.65, *p* < 0.0001). Diastolic (DBP) and systolic blood pressure (SBP) both had higher values in patients with steatosis (79.06 ± 7.76 vs. 76.87 ± 7.62 and 127.01 ± 14.44 vs. 124.30 ± 15.28, *p* < 0.0001 and *p* = 0.001, respectively), while there were no differences for diabetes and hypertension (*p* = 0.25 and *p* = 0.29, respectively). In line with the literature, there were significantly more patients with metabolic syndrome (MetS) and steatosis (38.53% vs. 20.57%, *p* < 0.001).

Blood parameters were analyzed, and some were found to be significant. Steatosis patients had higher levels of glucose, triglycerides, insulin, HOMA-IR (homeostasis model assessment-estimated insulin resistance), hemoglobin, MCHC (mean corpuscular hemoglobin concentration), WBCs (white blood cells), neutrophils, lymphocytes, monocytes, HbA1c (hemoglobin A1c), SGPT (serum glutamic pyruvic transaminase), GGT (gamma-glutamyl transferase), creatinine, FT3 (free triiodothyronine), and CRP (C-reactive protein), with all statistical significance at *p* < 0.05. In line with the disease condition, steatosis generated lower levels of HDL (high-density lipoprotein), RBCs (red blood cells), MCV (mean corpuscular volume), basophils, and GOT (aspartate aminotransferase), with *p* < 0.05.

Table 2 shows the mean consumption of food groups. Intake differences were found between groups. More red meat (25.05 ± 22.76 vs. 23.32 ± 31.80, *p* = 0.001), processed meat (4.87 ± 6.84 vs. 4.48 ± 9.96, *p* = 0.03), ready-to-eat dishes (37.75 ± 38.04 vs. 35.10 ± 50.46, *p* = 0.01), and alcohol (both wine and beer) (122.97 ± 157.06 vs. 95.58 ± 139.01, *p* = 0.03; 45.09 ± 108.38 vs. 22.10 ± 57.91, *p* = 0.03) were consumed by steatotic subjects, and the difference was statistically significant. There was a lower intake of root vegetables (13.07 ± 26.23 vs. 15.37 ± 25.36, *p* = 0.01) and nuts (3.31 ± 6.04 vs. 3.93 ± 6.67, *p* = 0.04).

**Figure 2:**
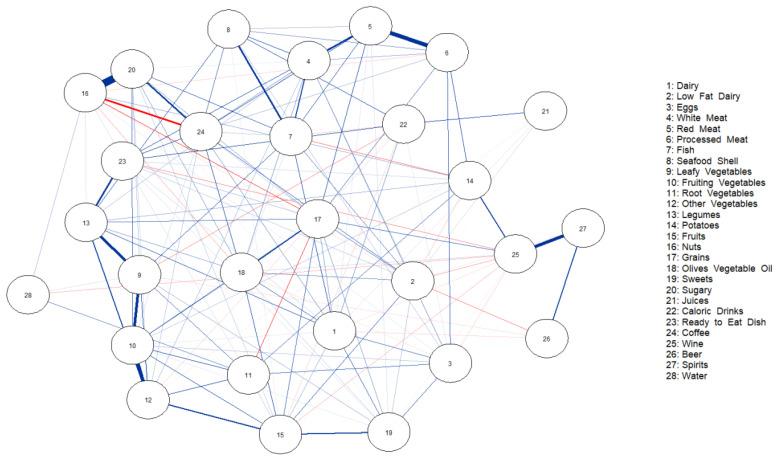
Network of partial correlations between food group intakes and additive intakes in patients without steatosis.

**Figure 3:**
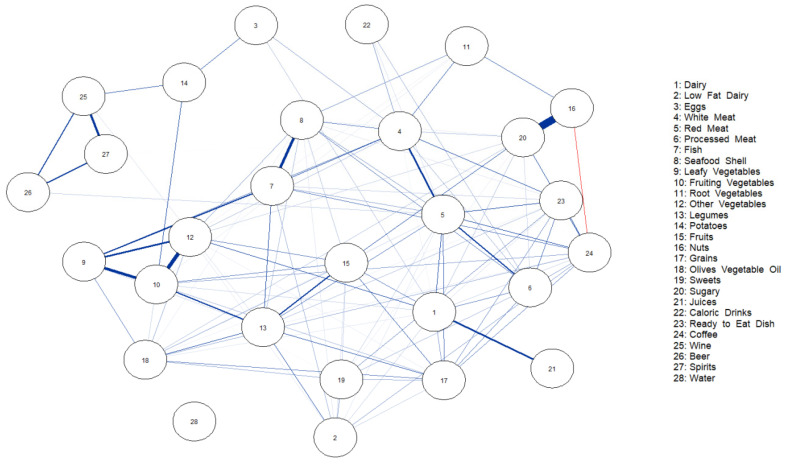
Network of partial correlations between food group intakes and additive intakes in patients with steatosis.

Figure 2 and Figure 3 show the network of dietary intake for non-obese patients without and with steatosis. The nodes represent the food groups, and the edges, the conditional dependencies between food groups (nodes). Blue lines show positive partial correlations, and red lines, negative correlations. The different thicknesses of the edges are related to the strengths of the correlations. Figure 2 shows the strongest partial correlation with nuts and sugars (0.81), followed by red meat and processed meat (0.49), and fruiting vegetables and other vegetables (0.43).

In the same way, in Figure 3, nuts and sugars had the strongest correlation (0.71), followed by fruiting vegetables and other vegetables (0.39), and leafy vegetables and fruiting vegetables (0.32).

Other correlation values are reported in the Supplementary Materials (Supplementary Tables S2 and S3), and the importance of each node is reported in a centrality plot (Supplementary Figure S1).

## 4. Discussion

The aim of this study was to examine a small cohort of apparently healthy participants living in Southern Italy, found to be characterized by a high prevalence of steatosis. The differences between blood parameters and dietary habits were investigated. A higher prevalence of steatosis in males had already been previously demonstrated in several cohorts [54], and this trend was confirmed in our non-obese cohort. BMI was higher in the cohort of cases, as amply demonstrated in the literature [55], but surprisingly, nearly 30% of our cohort suffered from metabolic syndrome. The blood profile of these patients was shown to be comparable to that of obese patients, with statistically higher levels of glucose, triglycerides, insulin, HOMA-IR, and hemoglobin [56].

These findings, together with the lack of difference in dietary intake between non-obese individuals with and without steatosis, indicated that non-obese steatotic patients may have other metabolic abnormalities that produce liver disease, and not only at a younger age. Other postulated causes could include a genetic predisposition, intestinal dysmotility, and other metabolic abnormalities not associated with weight gain [57].

Lean steatosis, classically described in Asian populations, has also been described in other populations in the Americas and Europe, with an incidence of 8–20% [58,59].

It is generally thought that thin patients have a different health awareness compared to obese patients, which is reflected in their choice of foods. In our case, weight was independent of food choices; in fact, we found very similar eating habits between the two groups. Although there was a greater daily consumption among steatotic patients of foods such as red meats and processed meats, together with unhealthy food and alcohol, and lower consumption of vegetables and fruits than in non-obese patients, a network analysis demonstrated that the variety of foods, as well as their combinations, were very similar between the two groups. Moreover, taste testing results revealed that overweight/obese participants liked both healthy and less healthy foods, as well as other food categories [60].

A liking for fatty foods is genetic, which may be due to multiple reasons, including their orosensory properties and post-ingestive and metabolic effects [61]. Fat is a concentrated source of energy with rewarding post-ingestive effects [60]. Sweet and salty high-fat foods have been proven to be particularly palatable [62,63]. Based on the hypothesis of a sweet tooth, several studies have shown that the liking of foods is not substantially different between obese and non-obese individuals, and reported that obese individuals liked sweet foods as much as, but perhaps even less than, non-obese individuals [64,65,66,67].

## 5. Conclusions

In conclusion, non-obese steatotic patients might have a distinct pathophysiology as compared to their more obese counterparts. We could hypothesize that BMI is just a marker to define a wide range of obesity. Non-obese steatosis subjects probably have compensatory physiological mechanisms that prevent weight gain but may equally develop severe and progressive liver disease [5,68,69]. However, these hypotheses need to be confirmed with future studies and analysis of associations also including the microbiota.

Steatosis has predominantly been associated with obesity and other metabolic conditions. However, its prevalence in lean or non-obese individuals is rising worldwide, underlining the importance of understanding the differences in physiological profiles among patients with different BMI. Patients with steatosis, regardless of the BMI, have an associated genetic predisposition, increased visceral adiposity, insulin resistance, poor eating habits, and little exercise. Furthermore, these patients show a higher prevalence of MetS than patients without steatosis. Lifestyle modifications remain the first line of treatment in steatosis, regardless of the BMI. In the future, it would be desirable to optimize clinical practice and decision-making for these apparently healthy patients, using the Delphi technique to collect opinions on particular food habits and clinical conditions [70]. Furthermore, the use of liver biopsy or DNA sequencing methodologies could explain the obtained results. The role of emerging therapeutics in lean or non-obese steatosis patients is unclear, and further studies are necessary.

Confounding factors, such as alcohol intake and weight loss following disease progression, could explain the more severe liver disease and a worse outcome in some patients with lean or non-obese steatosis, and genetic factors are increasingly recognized to play an important role. Further studies to understand the genetic determinants in these patients with steatosis could open the door to better diagnostics and therapeutics that may have the potential to be expanded to obese steatosis patients.

## Figures and Tables

**Table 1:** Epidemiological and clinical characteristics of non-obese patients (BMI < 30 kg/m^2^) with and without steatosis. MICOL cohort (*n* = 987).

Parameters *	Total Cohort (*n = 987*)	Steatosis		*p* ^^^
		No (*n = 564*)	Yes (*n = 423*)	
Gender (M) (%)	579 (58.66)	290 (51.42)	289 (68.32)	<0.001 ^ѱ^
Age (years)	63.56 ± 11.57	63.72 ± 12.22	63.34 ± 10.66	0.68
Degree of Education (%)				0.11 ^ѱ^
No	262 (28.20)	160 (30.02)	102 (25.76)	
Elementary School	295 (31.75)	151 (28.33)	144 (36.36)	
Secondary School	245 (26.37)	147 (27.58)	98 (24.75)	
High School	83 (8.93)	51 (9.57)	32 (8.08)	
Short Degree	44 (4.74)	24 (4.50)	20 (5.05)	
Smoker (Yes) (%)	139 (14.96)	81 (15.53)	58 (14.61)	0.79 ^ѱ^
BMI (Kg/m^2^)	25.66 ± 2.69	24.87 ± 2.65	26.72 ± 2.37	<0.0001
DBP (mmHg)	77.81 ± 7.75	76.87 ± 7.62	79.06 ± 7.76	<0.0001
SBP (mmHg)	125.30 ± 15.28	124.02 ± 15.76	127.01 ± 14.44	0.001
Diabetes (Yes) (%)	66 (8.71)	34 (7.71)	32 (10.09)	0.25 ^ѱ^
Hypertension (Yes) (%)	345 (45.39)	194 (43.79)	151 (47.63)	0.29 ^ѱ^
MetS (Yes) (%)	279 (28.27)	116 (20.57)	163 (38.53)	<0.001 ^ѱ^
*Blood Parameters*				
Glucose (mg/dL)	97.84 ± 20.86	94.69 ± 16.43	102.04 ± 25.01	<0.0001
Cholesterol (mg/mL)	193.26 ± 37.67	192.18 ± 37.78	194.71 ± 37.52	0.35
HDL (mg/dL)	50.95 ± 13.24	53.39 ± 13.34	47.71 ± 12.40	<0.0001
LDL (mg/dL)	122.95 ± 33.05	122.17 ± 32.62	123.92 ± 33.61	0.31
Triglycerides (mg/dL)	98.94 ± 57.79	86.25 ± 47.72	115.81 ± 65.28	<0.0001
Insulin (U/L)	8.10 ± 27.48	7.71 ± 35.99	8.63 ± 5.98	<0.0001
HOMA-IR	2.12 ± 8.84	2.01 ± 11.56	2.26 ± 2.11	<0.0001
RBCs (M/mcL)	4.87 ± 0.52	4.81 ± 0.53	4.95 ± 0.49	<0.0001
Hemoglobin (g/dL)	14.06 ± 1.49	13.85 ± 1.51	14.35 ± 1.42	<0.0001
HCT (%)	42.41 ± 3.38	41.96 ± 3.40	42.94 ± 3.29	0.0003
MCV (fL)	85.56 ± 6.81	85.75 ± 7.29	85.34 ± 6.19	0.04
MCH (pg)	28.92 ± 2.57	28.81 ± 2.72	29.05 ± 2.37	0.25
MCHC (g/dL)	33.79 ± 1.15	33.58 ± 1.13	34.03 ± 1.12	<0.0001
RDW-CV (%)	13.63 ± 1.16	13.67 ± 1.24	13.60 ± 1.06	0.78
Platelets (K/mcL)	228.90 ± 59.83	230.44 ± 62.35	226.85 ± 56.32	0.39
WBCs (K/mcL)	5.98 ± 2.11	5.83 ± 2.19	6.17 ± 1.98	0.0001
Neutrophils (%)	57.05 ± 8.65	57.35 ± 8.72	56.69 ± 8.56	0.23
Lymphocytes (%)	32.20 ± 8.27	31.99 ± 8.39	32.45 ± 8.13	0.37
Eosinophils (%)	2.88 ± 1.82	2.88 ± 1.83	2.88 ± 1.80	0.96
Monocytes (%)	7.34 ± 1.84	7.21 ± 1.73	7.59 ± 1.95	0.07
Basophils (%)	0.52 ± 0.30	0.56 ± 0.34	0.48 ± 0.24	0.05
Neutrophils (10^3^/µL)	3.44 ± 1.46	3.34 ± 1.17	3.57 ± 1.75	0.04
Lymphocytes (10^3^/µL)	1.95 ± 1.71	1.90 ± 2.08	2.01 ± 1.12	0.0004
Monocytes (10^3^/µL)	0.43 ± 0.17	0.41 ± 0.16	0.46 ± 0.17	0.0001
Eosinophils (10^3^/µL)	0.17 ± 0.11	0.16 ± 0.11	0.17 ± 0.11	0.15
Basophils (10^3^/µL)	0.03 ± 0.02	0.03 ± 0.02	0.03 ± 0.01	0.87
HbA1c (mmol/mol)	36.56 ± 7.43	35.10 ± 6.33	38.30 ± 8.23	<0.0001
Fractional total bilirubinemia (mg/dL)	0.72 ± 0.37	0.71 ± 0.38	0.74 ± 0.37	0.32
Direct fractional bilirubinemia (mg/dL)	0.16 ± 0.05	0.16 ± 0.05	0.16 ± 0.05	0.24
Indirect fractional bilirubinemia (mg/dL)	0.50 ± 0.28	0.46 ± 0.24	0.55 ± 0.33	0.33
GOT (U/L)	22.90 ± 20.08	23.14 ± 25.50	22.58 ± 8.70	0.01
SGPT (U/L)	22.68 ± 18.08	21.61 ± 21.25	24.10 ± 12.56	<0.0001
GGT (U/I)	19.71 ± 15.83	17.89 ± 13.20	22.14 ± 18.49	<0.0001
Albumin (%)	4.15 ± 0.26	4.13 ± 0.26	4.17 ± 0.25	0.10
Iron (mg/dL)	90.02 ± 30.94	88.76 ± 30.97	91.52 ± 30.90	0.35
Urea (mg/dL)	40.02 ± 10.80	40.18 ± 11.87	39.80 ± 9.19	0.71
Creatinine (mg/dL)	0.82 ± 0.34	0.81 ± 0.42	0.83 ± 0.18	0.001
eGFR (mL/min)	84.97 ± 9.98	85.47 ± 10.41	84.39 ± 9.44	0.14
AAT (mg/dL)	184.50 ± 40.52	183.68 ± 39.58	185.48 ± 41.67	0.78
Folate (ng/mL)	8.46 ± 4.95	8.64 ± 4.97	8.23 ± 4.91	0.08
Vitamin B12 (pg/mL)	368.02 ± 513.64	385.44 ± 575.76	344.86 ± 416.57	0.64
TSH (mUI/mL)	959.08 ± 1395.93	961.07 ± 1501.29	956.44 ± 1245.06	0.89
FT3 (pg/mL)	3.32 ± 0.47	3.29 ± 0.45	3.36 ± 0.48	0.01
FT4 (ng/mL)	0.87 ± 0.32	0.86 ± 0.15	0.87 ± 0.45	0.52
CRP (mg/L)	0.23 ± 0.49	0.17 ± 0.26	0.30 ± 0.65	<0.0001

* As mean and standard deviation for continuous variables and as frequency and percentage (%) for categorical variables. ^^^ Wilcoxon rank-sum test (Mann–Whitney), ^ѱ^ chi-square test. Abbreviations: DBP, diastolic blood pressure; SBP, systolic blood pressure; BMI, body mass index; MeS, metabolic syndrome; HDL, high-density lipoprotein; LDL, low-density lipoprotein; HOMA-IR, homeostasis model assessment-estimated insulin resistance; RBCs, red blood cells; HCT, hematocrit (he-MAT-uh-krit); MCV, mean corpuscular volume; MCH, mean corpuscular hemoglobin; MCHC, mean corpuscular hemoglobin concentration; RDW-CV, red cell distribution width; WBCs, white blood cells; HbA1c, hemoglobin A1c; GOT, aspartate aminotransferase; SGPT, serum glutamic pyruvic transaminase; GGT, gamma-glutamyl transferase; eGFR, estimated glomerular filtration rate; AAT, alpha-1-antitrypsin; TSH, thyroid-stimulating hormone; FT3, free triiodothyronine; FT4, thyroxine; PCR, CRP, C-reactive protein.

**Table 2:** Dietary daily intake of 28 food groups among steatotic and healthy non-obese patients of MICOL 4.

Food Groups *	Total Cohort	Steatosis		*p* ^^^
		No	Yes	
Dairy	76.30 ± 30	70.63 ± 92.14	83.87 ± 114.95	0.06
Low-Fat Dairy	66.17 ± 98.33	65.28 ± 98.41	67.36 ± 98.32	0.39
Eggs	9.19 ± 8.12	9.39 ± 8.50	8.93 ± 7.59	0.37
White Meat	20.97 ± 26.32	20.56 ± 25.27	21.51 ± 27.68	0.79
Red Meat	24.06 ± 28.28	23.32 ± 31.80	25.05 ± 22.76	0.01
Processed Meat	4.64 ± 8.76	4.48 ± 9.96	4.84 ± 6.84	0.03
Fish	19.07 ± 21.56	18.89 ± 22.41	19.31 ± 20.41	0.22
Seafood/Shellfish	4.10 ± 6.25	3.98 ± 6.49	4.25 ± 5.94	0.08
Leafy Vegetables	44.36 ± 58.07	45.37 ± 62.00	43.01 ± 52.40	0.82
Fruiting Vegetables	70.43 ± 81.68	69.30 ± 83.64	71.93 ± 79.08	0.33
Root Vegetables	14.38 ± 25.75	15.37 ± 25.36	13.07 ± 26.23	0.01
Other Vegetables	63.19 ± 82.08	65.58 ± 87.62	60.02 ± 74.06	0.80
Legumes	26.08 ± 29.67	26.03 ± 31.32	26.15 ± 27.35	0.81
Potatoes	12.99 ± 16.71	13.04 ± 15.97	12.94 ± 17.65	0.59
Fruits	360.35 ± 443.83	353.10 ± 445.82	370.01 ± 441.49	0.32
Nuts	3.66 ± 6.41	3.93 ± 6.67	3.31 ± 6.04	0.04
Grains	118.61 ± 122.41	113.64 ± 120.94	125.23 ± 124.17	0.09
Olives and Vegetable Oil	32.74 ± 32.04	31.40 ± 28.02	34.53 ± 36.68	0.18
Sweets	19.91 ± 39.50	19.79 ± 41.09	20.07 ± 37.33	0.49
Sugars	13.81 ± 19.97	14.16 ± 20.28	13.35 ± 19.56	0.71
Juices	8.89 ± 21.56	8.58 ± 19.40	9.32 ± 24.15	0.35
High-Calorie Drinks	9.86 ± 30.12	8.84 ± 25.51	11.22 ± 35.32	0.16
Ready-to-Eat Dishes	36.22 ± 45.55	35.10 ± 50.46	37.72 ± 38.04	0.01
Coffee	45.79 ± 41.65	43.98 ± 40.15	48.22 ± 43.50	0.15
Wine	107.32 ± 139.01	95.58 ± 122.60	122.97 ± 157.06	0.03
Beer	31.95 ± 84.09	22.10 ± 57.91	45.09 ± 108.38	0.03
Spirits	1.75 ± 4.93	1.48 ± 4.39	2.10 ± 5.55	0.22
Water	658.39 ± 269.12	667.34 ± 278.95	646.45 ± 255.27	0.09

* As mean and standard deviation (M ± SD). Food groups were calculated on the quantity of daily consumption (grams). ^^^ Wilcoxon rank-sum test (Mann–Whitney).

## Data Availability

The original contributions presented in the study are included in the article. Further inquiries can be directed to the corresponding author.

## Supplementary Materials

The following supporting information can be downloaded at: https://www.mdpi.com/article/10.3390/nu15122713/s1, Table S1: Single foods from the questionnaire relative to the food groupings used for the analyses; Table S2: Partial matrix correlation of every pair of food groups, in non-obese patients without steatosis; Table S3: Partial matrix correlation of every pair of food groups, in non-obese patients with steatosis; Figure S1: (A) Centrality plot for food group daily intakes, in lean patients without steatosis. (B) Centrality plot for food group daily intakes, in lean patients with steatosis.
