# Supplementary material for: Food Network Analysis in Non-Obese Patients with or without Steatosis
Source: Nutrients. 2023 Jun 11;15(12):2713. doi: 10.3390/nu15122713 (PMC10301509; doi:10.3390/nu15122713)
Supplement: Supplementary file 1 [file nutrients-15-02713-s001.zip › nutrients-2381694-Supplementary Material.pdf]

nutrients MDPI

Supplementary Materials

Table S1. Single food from the questionnaire and relative the food grouping used for the analyses.

Food Group	Sample foods
1) Dairy	Whole Milk; Yogurt; Mozzarella Cheese; Scamorza; Fresh Caciottina-Stracchino-Fontina; Bel Paese-Gorgonzola Cheese; Provolone-Caciocavallo; Grana Padano-Parmesan; Pecorino Cheese-Vacchino; Swiss Cheese; Cheese Spread; Ice Cream
2) Low Fat Dairy	Skimmed-Partly Skimmed Milk; Cottage Cheese
3) Eggs	Eggs
4) White Meat	Chicken; Rabbit
5) Red Meat	Veal; Horse; Pork; Liver; Lamb
6) Processed Meat	Fresh Sausage; Raw Ham; Mortadella-Cooked Ham; Salami
7) Fish	Sole-Sea Bream-Snapper-Sea Bass-Grouper; Codfish-Stingray-Dogfish; Goatfish-Mullet-Mackerel; Anchovies-Sardines; Tuna in Oil
8) Sea Food/Shellfish	Octopus-Cuttlefish-Squid-Prawns; Mussels-Other Seafoods
9) Leafy Vegetables	Spinach-Chard-Chicory-Salad
10) Fruiting Vegetables	Artichokes; Zucchini-Eggplants; Tomatoes; Cucumbers; Peppers
11) Root Vegetables	Carrots
12) Other Vegetables	Cabbage-Cauliflower-Broccoli-Green Turnips; Vegetable Soup; Fennels-Celery
13) Legumes	Chickpeas-Lentils-Beans; Peas; Green Beans; Broad Beans with Vegetables
14) Potatoes	Potatoes
15) Fruits	Oranges-Tangerines-Grapefruits; Peaches; Figs; Apricots; Grapes; Watermelon; Melon; Apples-Pears; Kiwi; Cherries; Banane
16) Nuts	Nuts
17) Grains	Pasta; Rice or Risotti; Pasta or Rice in Broth; Bread
18) Olives and Vegetable Oil	Olives; Olive Oil
19) Sweets	Sweets; Chocolate; Pastries; Cookies-Biscuits
20) Sugary	Sugar; Fruit in Syrup
21) Juices	Fruit Juice
22) Caloric Drinks	Coke-Orange-Juice-Chinotto
23) Ready to Eat Dish	Pizza; Focaccia
24) Coffee	Coffee; Barley Coffee
25) Wine	Wine
26) Beer	Beer
27) Spirits	Liquor
28) Water	Water

Table S2. Partial matrix correlation of every pair of food groups, in non-obese patients without steatosis

Food-Groups	1	2	3	4	5	6	7	8	9	10	11	12	13	14	15	16	17	18	19	20	21	22	23	24	25	26	27	28
**1**	--																											
**2**	0.00	--																										
**3**	0.07	0.00	--																									
**4**	0.00	0.03	0.06	--																								
**5**	0.01	0.00	0.00	0.24	--																							
**6**	0.00	0.00	0.08	0.04	0.49	--																						
**7**	0.11	0.07	0.00	0.17	0.14	0.00	--																					
**8**	0.00	0.00	0.03	0.13	0.00	0.05	0.20	--																				
**9**	0.00	0.00	0.00	0.00	0.00	0.00	0.13	0.00	--																			
**10**	0.01	0.00	0.03	0.00	0.00	0.00	0.00	0.00	0.30	--																		
**11**	0.00	0.00	0.07	0.00	0.00	0.00	0.00	0.00	0.08	0.11	--																	
**12**	0.00	0.00	0.00	0.04	0.00	0.00	0.00	0.00	0.09	0.43	0.10	--																
**13**	0.09	0.00	0.00	0.03	0.00	0.00	0.00	0.13	0.30	0.00	0.00	0.00	--															
**14**	0.00	-0.02	0.00	0.00	0.00	0.13	-0.04	0.00	0.00	0.03	0.08	0.00	0.04	--														
**15**	0.04	0.09	0.00	0.00	0.00	0.00	0.00	0.01	0.00	0.13	0.03	0.17	0.00	0.00	--													
**16**	0.00	-0.01	0.01	0.00	0.00	-0.02	0.00	0.00	0.00	0.00	0.02	0.00	-0.02	0.04	0.00	--												
**17**	0.05	0.07	0.00	0.00	0.11	0.08	0.00	0.00	0.00	0.05	-0.08	0.00	0.07	0.00	0.08	-0.09	--											
**18**	0.08	0.06	-0.01	0.05	0.00	0.00	0.01	0.00	0.00	0.10	0.00	0.00	0.00	0.00	0.11	-0.04	0.19	--										
**19**	0.08	0.03	0.14	0.00	0.01	0.00	0.00	0.00	0.04	0.01	0.00	0.00	0.00	0.00	0.17	0.00	0.05	0.03	--									
**20**	0.00	0.00	0.00	0.01	0.00	0.00	0.08	0.00	0.03	0.08	0.00	0.02	0.00	0.00	0.03	0.81	0.05	0.05	0.01	--								
**21**	0.01	0.02	0.00	0.00	0.00	0.00	0.00	0.00	0.00	0.00	0.00	-0.01	0.00	0.00	0.00	0.00	0.00	0.00	0.00	0.00	--							
**22**	0.05	0.00	0.00	0.00	0.00	0.00	-0.02	0.11	-0.05	0.00	0.00	-0.02	0.00	0.00	-0.02	0.00	0.00	-0.01	0.00	0.00	0.10	--						
**23**	0.02	0.03	0.00	0.00	0.11	0.00	0.00	0.00	0.00	0.00	0.00	0.00	0.21	0.02	0.00	0.00	0.03	0.00	0.02	0.00	0.00	0.09	--					
**24**	0.06	0.08	0.00	0.06	0.09	0.03	0.00	0.00	0.00	0.02	0.00	0.03	0.00	0.00	0.00	-0.19	0.03	0.00	0.00	0.21	0.00	0.00	0.14	--				
**25**	-0.01	-0.04	-0.02	0.00	0.00	0.00	0.00	0.00	0.00	0.00	0.00	0.00	0.00	0.17	0.00	0.00	0.07	0.02	-0.01	0.00	0.00	0.00	-0.05	0.00	--			
**26**	-0.02	-0.05	0.00	0.00	0.00	0.00	0.00	0.00	-0.01	0.00	0.00	0.00	0.00	0.00	0.00	0.00	0.00	0.00	0.00	0.00	0.00	0.00	0.00	0.00	0.00	--		
**27**	0.00	-0.01	0.00	0.00	0.00	0.00	0.00	0.00	0.00	0.00	0.00	0.00	0.00	0.00	0.00	0.00	0.00	0.00	0.00	0.00	0.00	0.00	0.00	0.00	0.39	0.16	--	
**28**	0.00	0.00	0.00	0.00	0.00	0.00	0.00	0.00	0.01	0.00	0.06	0.00	0.00	0.00	0.00	0.00	0.00	0.00	0.00	0.00	0.00	0.00	0.00	0.00	-0.03	0.00	0.00	--

Table S3. Partial matrix correlation of every pair of food groups, in non-obese patients with steatosis.

Food-Groups	**1**	**2**	**3**	**4**	**5**	**6**	**7**	**8**	**9**	**10**	**11**	**12**	**13**	**14**	**15**	**16**	**17**	**18**	**19**	**20**	**21**	**22**	**23**	**24**	**25**	**26**	**27**	**28**
**1**	--																											
**2**	0.03	--																										
**3**	0.04	0.00	--																									
**4**	0.01	0.00	0.06	--																								
**5**	0.12	0.02	0.00	0.23	--																							
**6**	0.08	0.00	0.00	0.05	0.15	--																						
**7**	0.00	0.05	0.00	0.13	0.05	0.00	--																					
**8**	0.00	0.01	0.00	0.08	0.06	0.09	0.30	--																				
**9**	0.00	0.00	0.00	0.08	0.00	0.00	0.15	0.00	--																			
**10**	0.02	0.00	0.00	0.00	0.00	0.00	0.00	0.00	0.32	--																		
**11**	0.00	0.00	0.00	0.13	0.00	0.00	0.00	0.07	0.00	0.01	--																	
**12**	0.11	0.01	0.00	0.04	0.00	0.00	0.00	0.06	0.23	0.39	0.01	--																
**13**	0.00	0.13	0.00	0.00	0.00	0.00	0.12	0.04	0.02	0.17	0.00	0.00	--															
**14**	0.00	0.00	0.11	0.00	0.00	0.00	0.00	0.00	0.00	0.12	0.00	0.00	0.00	--														
**15**	0.08	0.02	0.00	0.00	0.01	0.00	0.00	0.03	0.00	0.07	0.00	0.08	0.17	0.00	--													
**16**	0.00	0.00	0.00	0.00	0.00	0.00	0.00	0.00	0.00	0.00	0.09	0.00	0.00	0.00	0.00	--												
**17**	0.06	0.03	0.00	0.00	0.09	0.09	0.00	0.00	0.00	0.04	0.00	0.01	0.10	0.00	0.12	0.00	--											
**18**	0.01	0.00	0.00	0.00	0.00	0.00	0.00	0.01	0.08	0.02	0.00	0.05	0.14	0.00	0.05	0.00	0.08	--										
**19**	0.00	0.06	0.00	0.00	0.03	0.04	0.00	0.00	0.00	0.00	0.00	0.00	0.00	0.00	0.03	0.00	0.05	0.05	--									
**20**	0.04	0.00	0.00	0.04	0.00	0.02	0.00	0.01	0.00	0.00	0.00	0.03	0.00	0.00	0.14	0.71	0.00	0.04	0.01	--								
**21**	0.19	0.00	0.00	0.00	0.00	0.00	0.00	0.00	0.00	0.00	0.00	0.00	0.00	0.00	0.00	0.00	0.00	0.00	0.00	0.00	--							
**22**	0.00	0.00	0.00	0.06	0.02	0.05	0.00	0.00	0.00	0.00	0.00	0.00	0.00	0.00	0.00	0.00	0.00	0.00	0.00	0.00	0.00	--						
**23**	0.02	0.00	0.00	0.11	0.00	0.09	0.03	0.01	0.00	0.00	0.00	0.00	0.04	0.00	0.00	0.00	0.07	0.01	0.07	0.00	0.00	0.00	--					
**24**	0.02	0.00	0.00	0.00	0.12	0.06	0.08	0.00	0.00	0.05	0.00	0.00	0.01	0.00	0.00	-0.09	0.08	0.05	0.00	0.14	0.00	0.00	0.13	--				
**25**	0.00	0.00	0.00	0.00	0.00	0.00	0.00	0.00	0.00	0.00	0.00	0.00	0.02	0.10	0.00	0.00	0.00	0.00	0.00	0.00	0.00	0.00	0.00	0.00	--			
**26**	0.00	0.00	0.00	0.00	0.03	0.00	0.00	0.00	0.00	0.00	0.00	0.00	0.00	0.00	0.00	0.00	0.00	0.00	0.00	0.00	0.00	0.00	0.00	0.00	0.17	--		
**27**	0.00	0.00	0.00	0.00	0.00	0.00	0.00	0.00	0.00	0.00	0.00	0.00	0.00	0.00	0.00	0.00	0.00	0.00	0.00	0.00	0.00	0.00	0.00	0.00	0.24	0.18	--	
**28**	0.00	0.00	0.00	0.00	0.00	0.00	0.00	0.00	0.00	0.00	0.00	0.00	0.00	0.00	0.00	0.00	0.00	0.00	0.00	0.00	0.00	0.00	0.00	0.00	0.00	0.00	0.00	--

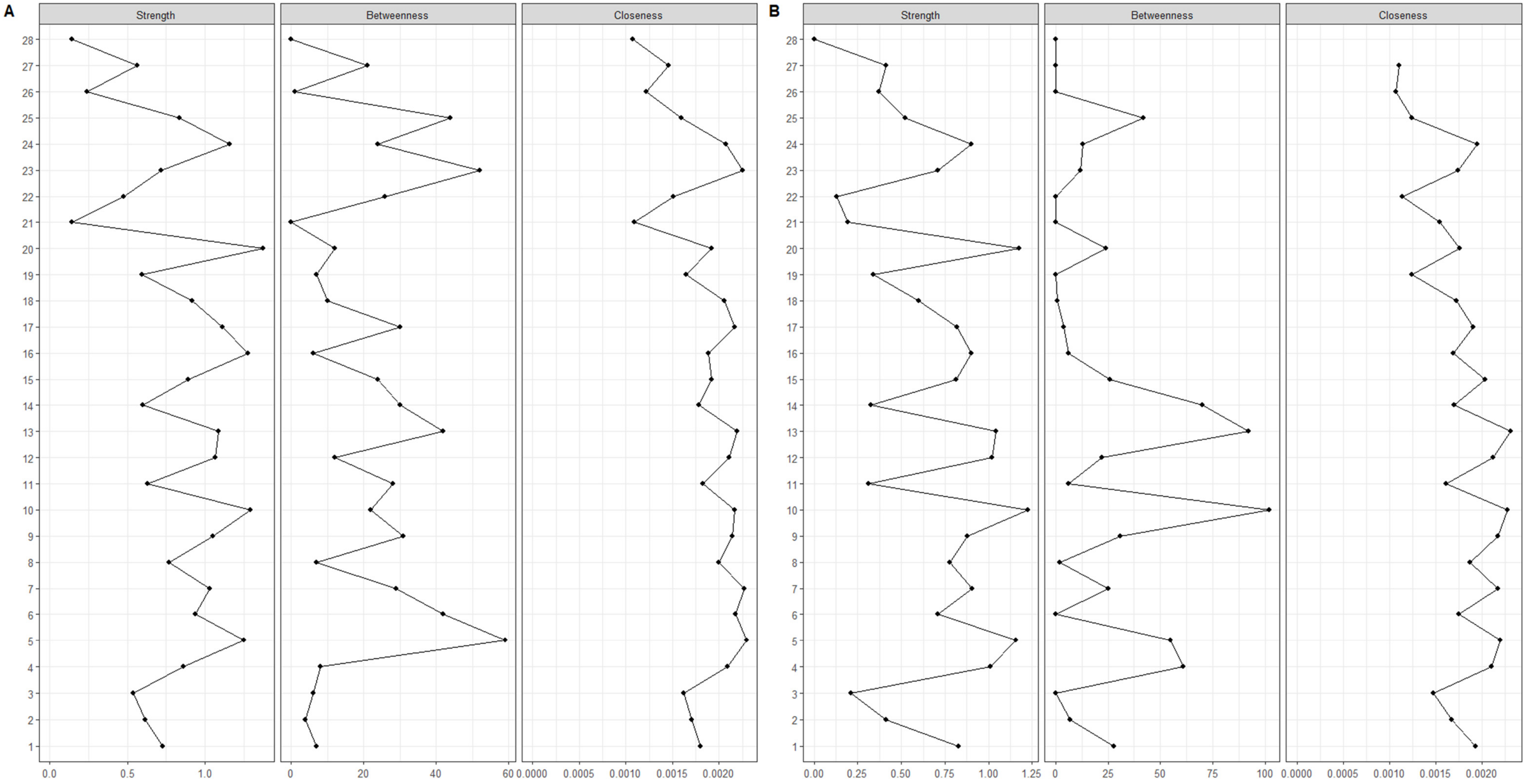

Figure S1. (A) Centrality plot for food group daily intake, in lean patients without steatosis. (B) Centrality plot for food group daily intake, in lean patients with steatosis.
